# Strategies to Reduce Mental Illness Stigma: Perspectives of People with Lived Experience and Caregivers

**DOI:** 10.3390/ijerph19031632

**Published:** 2022-01-31

**Authors:** Shazana Shahwan, Chong Min Janrius Goh, Gregory Tee Hng Tan, Wei Jie Ong, Siow Ann Chong, Mythily Subramaniam

**Affiliations:** Research Division, Institute of Mental Health, 10 Buangkok View, Singapore 539747, Singapore; janrius_cm_goh@imh.com.sg (C.M.J.G.); gregtth@gmail.com (G.T.H.T.); wei_jie_ong@imh.com.sg (W.J.O.); siow_ann_chong@imh.com.sg (S.A.C.); mythily@imh.com.sg (M.S.)

**Keywords:** mental health, stigma, discrimination, contact, attitudes, behavior, anti-stigma

## Abstract

Background: Reducing the stigma surrounding mental illness is a global public health priority. Traditionally, anti-stigma campaigns were led by mental health professionals/organisations and had an emphasis on increasing mental health literacy. More recently, it has been argued that people with lived experience have much to contribute in terms of extending and strengthening these efforts. The purpose of this paper was to elicit views and suggestions from people with lived experience (PWLE) as well as from informal caregivers of people with mental health conditions, on effective strategies to combat the stigma surrounding mental illness. Methods: Six focus group discussions (FGDs) were carried out with PWLE recruited at outpatient services at the Institute of Mental Health, Singapore, and five FGDs were carried out with informal caregivers who responded to advertisements for the study between March and November 2018. In all, the sample comprised 42 PWLE and 31 caregivers. All the FGDs were transcribed verbatim and were analysed using thematic analysis. A pragmatic approach was adopted for the study, and the researchers did not assume any particular philosophical orientation. Results: Four overarching themes depicting strategies to combat stigma were identified through thematic analysis. They were (1) raising mental health awareness, (2) social contact, (3) advocacy by influential figures or groups, and (4) the legislation of anti-discriminatory laws. Conclusions: These strategies were in line with approaches that have been used internationally to disrupt the process of stigma. Our study has further identified nuanced details on how these strategies can be carried out as well as possible areas of priority in the Singapore landscape.

## 1. Introduction

The stigma of living with a mental health condition has been described as being worse than the experience of the illness itself [1]. The aversive reactions that members of the general population have towards people with mental illness is known as public stigma and can be understood in terms of (i) stereotypes, (ii) prejudice, and (iii) discrimination [2]. Common stereotypes associated with people with mental health conditions are that they are dangerous, incompetent, and weak in character. Prejudice refers to the agreement with these stereotypes, while discrimination refers to behavioural reactions to these prejudices [3]. 

Beyond the interpersonal manifestations of public stigma towards people with mental health conditions, societal-level conditions such as institutional policies and practices and cultural norms have also been found to be biased against people with mental health conditions, resulting in a lack of opportunities and resources being afforded to them [4]. These socio-political disinclinations, known as structural stigma, result in people with mental health conditions being excluded from employment, living in unstable and unsafe conditions, being disqualified from health insurance, and being subjected to coercive hospitalisation and treatment [5,6]. The deprivation of opportunities and poor-quality resources provided to those with mental health conditions have severe bearings, as evidenced by the gross overrepresentation of individuals with mental health conditions in the criminal justice system and among those living in poverty [7]. People with mental health conditions also have significantly higher morbidity and mortality rates [8], and consequent to all the above, have a lower quality of life compared to the general population [9,10]. 

Through repeated encounters with public and structural stigma, individuals with mental health conditions are inclined to internalise these reactions, a phenomenon known as self-stigma. A systematic review found that exposure to public stigma predicts self-stigma at a later time [11,12]. A person’s own stigmatizing views towards mental illness is associated with lower readiness to appraise his or her own symptoms as potentially indicating a mental health problem and thus reduces help-seeking behaviour [13]. This could be because the individual seeks to avoid the label of mental illness for him- or herself [14], fathomably to guard themselves against the negative self-perceptions associated with it and the potential consequences of shame and reduced empowerment [15]. Indeed, self-stigma decreases one’s self-esteem and self-efficacy, leading to the “why try effect”, where people with mental health conditions question their worthiness and capability to pursue personal goals [16,17], leading to a loss of self-respect and increased shame and hopelessness [18,19]. Over time, higher levels of self-stigma have been found to be associated with suicidal ideation [18,20]. 

Due to these adverse effects of stigma, stigma-reduction is seen as a global public health priority [21,22,23,24]. Anti-stigma programmes were traditionally conducted by or in substantial consultation with groups representing psychiatric expertise [25]. However, several criticisms have been raised towards this approach in the recent years. First, the emphasis on medical understandings of mental health problems and the importance of adhering to psychiatric interventions have been criticised as fulfilling the psychiatric services agenda rather than the interest of people with mental health conditions and eclipsing inputs from other standpoints [3,26]. Next, mental health professionals have been found to be just as likely to stigmatise those with mental health conditions [27,28,29]. Thus, is has been argued that the professional expertise that mental health professionals have in providing mental health services may be insufficient in impacting the social spheres in which stigma operate, and it may be timely for them to move to a supporting role [30]. 

In recent years, anti-stigma programmes have involved people with lived experience to allow direct or parasocial interactions between target audiences and people with mental health conditions. Contact-based interventions have demonstrated the clearest evidence in reducing stigmatising attitudes, desire for social distancing and discrimination [31,32,33]. Contact-based interventions typically involve brief contact between members of a majority group and a stranger representing the stigmatized population who is quite different from a naturally occurring contact. Stigma is reduced by providing an opportunity for interpersonal contact between people who have mental illness and individuals who may hold stigma towards them. A key ingredient of contact-based interventions is the delivery of testimonies by service users [34]. The efficacy of contact-based education has led to calls for collaborations with individuals with mental health conditions to offer their experiential wisdom in challenging stigma, representing the voice of those who struggle with mental health difficulties and shedding light on blind spots and gaps in previous strategies [3,35]. Corrigan asserted that just as disability rights groups have adopted the slogan of “no policy or action should be taken about a group without full participation of that group”, the same should be applied to alleviate mental health stigma [30]. Additionally, while we have thus far described the negative processes that arise from stigma, there are people with mental health conditions who do not agree with the hackneyed stereotypes and respond with indignation that seems to empower and energise them to advocate for changes to the ways in which they are treated [2]. Their inputs towards initiatives that are aimed at improving service delivery and de-stigmatisation programmes have been found to lead to novel results and have been described as a strength of those programmes [35,36].

Singapore is a small, highly urbanised, multi-cultural country located at the Southern tip of the Malayan Peninsula. The resident population is made up of 75.9% Chinese, 15.0% Malay, 7.5% Indian, and 1.6% other ethnicities [37]. A developed country, the culture of Singapore can be described as a combination of Eastern and Western cultures, and English is the primary language of instruction. Stigma towards mental illness remains prevalent in Singapore today. An earlier nationwide survey revealed that 38.3% of the population believed that people with mental illness are dangerous, and 49.6% felt that people need to be protected from psychiatric patients [38]. A decade later, another population survey, which used a vignette-based approach, reported that 50.8% of respondents indicated that mental illness was a sign of personal weakness, 42.8% were unwilling to work closely with a person with mental health conditions on a job, and 70.2% were unwilling to have a person with mental health conditions marry into their family [39]. A recent qualitative study of daily encounters of personal stigma reported themes such as social exclusion, subjection to contemptuous treatment, and rejection by employers following the declaration of a mental health condition [40]. 

Anti-stigma activities in Singapore have been conducted by the state psychiatric institution, the Institute of Mental Health (IMH), the National Council of Social Service, the Health Promotion Board (statutory boards), and non-profit organisations such as the Singapore Association for Mental Health and Silver Ribbon Singapore, who have the collective aims of improving mental health literacy, access to mental health care, and improving the reintegration of people with mental health conditions into the community [41,42]. However, the involvement of individuals with mental health conditions in anti-stigma campaigns is lacking. The purpose of this paper was thus to elicit views and suggestions from people with lived experience (PWLE) and informal caregivers of people with mental health conditions on effective strategies to combat stigma.

## 2. Methods

The present study is part of a larger study that aimed to examine the nature of mental illness stigma in Singapore from the perspectives of five stakeholder groups, namely PWLE, informal caregivers, members of the general public, professionals working in mental health settings, and policy makers. The main purpose of this research was to provide actionable knowledge. It took a pragmatic approach common in health services research and did not assume any particular methodological orientation [43]. Only data from PWLE and caregivers were used in this analysis. The study was approved by the institutional ethics committee, the National Healthcare Group Domain Specific Review Board. Written informed consent was obtained from all participants before initiating study related procedures.

### 2.1. Participants

PWLE and caregivers were recruited through referrals by their clinicians or self-referral by learning about the study though poster advertisements placed in waiting areas at the IMH outpatient clinics. The IMH is Singapore’s largest provider of mental health care, providing pharmacological and psychosocial treatments as well as psychosocial rehabilitation for a range of subspecialties, including child and adolescent psychiatry, affective disorders, and psychosis. It has also spearheaded mental health education and anti-stigma events for the public. All the participants were required to be Singapore citizens and permanent residents, aged 21 years old and above, and could not be a student or professional from the mental health field. 

PWLE recruitment was limited to two types of psychiatric diagnoses, mood and psychotic disorders, to attain a more homogenous account of encounters with stigma. The groups were also separated by diagnosis to facilitate the identification of members in a group with each other and to provide comfort when expressing themselves. In all, six Focus Group Discussions (FGD) were conducted with PWLE between March to May 2018 (three with individuals with mood disorders, three with those with psychosis-related disorders). Referred and self-referred PWLE were deemed clinically stable by their treating clinicians and were able to provide informed consent. 

Although the poster advertisements indicated that the study sought caregivers of individuals with psychosis-related or mood disorders, no attempt was made to confirm the diagnosis of their care recipients with the treating clinicians. The caregiver group was independent of the PWLE group. Unlike the PWLE FGDs, the caregivers were not separated based on the diagnosis of their care recipient. As the initial FGDs did not identify any issue with this approach, the team carried out the rest of the FGDs in a similar manner. In all, five FGDs were conducted with caregivers between June and November 2018.

The PWLE FGDs ranged from 5–8 participants, while the caregiver FGDs ranged from 4–9 participants. The sociodemographic profiles of the PWLE and caregiver participants are presented in Table 1. Participants received an inconvenience fee at the end of the FGD. All FGDs were conducted in English.

### 2.2. Data Collection

The FGDs were conducted in a closed room that was relatively free from distractions in a community club, which was chosen because it is a neutral venue. Each FGD was conducted by two study team members, who served as the facilitator or the note taker for the day. The facilitators (either MS or SS) were trained and experienced in qualitative research methodologies. CMJG, OWJ, GTTH, SS, and MS took turns as note-takers in the different FGDs. 

After individual consent was taken to participate in the research and to audio-record the session, each participant filled out a sociodemographic form that collated information about age, gender, education level, ethnicity, and brief information about their illness (for PWLE) or caregiving relationship (for caregivers), and the completed form was returned to the facilitator. Participants were assured that all of the data collected from them would be kept confidential, the transcripts would be de-identified such that names and other identifying features would be omitted, the audio-recording would be deleted after transcription, and that there were no correct or incorrect answers before the discussion commenced. 

The experienced facilitators used a topic guide comprising open-ended questions that had been developed by the research team so that the data collected across the various FGDs would be as uniform as possible. Few specific questions were designed to elicit information that could be best addressed by a particular target group. The topic guide covered areas of mental illness stigma such as encounters of stigma and reasons for stigma. The team formulated the questions in a manner similar to that recommended by Krueger et al. [44], the recommendations of whom comprised the following: The questions should elicit information that directly relates to the study’s objectives. The questions should be easy for the participants to understand and should be phrased in a neutral manner so as not to bias participant responses. The questions can be answered by all the participants. Questions should be open-ended and not answered with a “yes” or “no” to facilitate descriptive responses. The questions should not make the participants uncomfortable when answering, and they should not trigger defensive responses. The team brainstormed the questions to answer the objectives of the research, and one researcher drafted the questioning route, rephrased, and reordered the questions to form a logical flow. The draft was circulated to the rest of the team, and suggestions were incorporated. The team aimed to keep the final total number of questions between 10–12. Decisions to omit questions were based on importance in addressing the research objectives, with final decisions being made by the lead investigator (MS). The questions were then tested out, using the first focus group as a pilot. The items that were used to elicit responses to the research question addressed in this paper was from the final segment of the topic guide: “How do you think stigma towards people with mental illness can be reduced” and “Have you heard of campaigns to reduce stigma towards those with mental illness? Is there anything that can be done better?”. The facilitator probed for range and depth of responses and sought clarification for responses that were unclear using neutral questions. Attempts were made to encourage responses from all members. The entire duration of each FGD lasted between 1.5–2 h. FGDS were carried out one at a time, first with the PWLE and then with the caregivers. At the end of each FGD, there was a debrief between the facilitator and note-taker, and a comprehensive summary was provided to the rest of the research team soon after to reflect on each session, to ensure that any problems were identified early and addressed, and emerging themes and unique points that had been raised were discussed. The FGDs were later transcribed verbatim for analysis. The decision was made by the team to cease data collection for PWLE and the caregiver groups when no new themes were identified, i.e., when data saturation was reached.

### 2.3. Analysis

The data were analysed using an inductive thematic analysis method [45]. Transcripts were first distributed amongst five study team members (SS, CMJG, GTTH, OWJ, and MS) for familiarisation with the collected data. Subsequently, each study team member independently identified preliminary codes from their respective transcripts. The study team members then came together, and through an iterative process of comparing the codes and combining, discarding, and redefining the codes, collaboratively decided on the final list of codes. A codebook was developed by the coders (SS, CMJG, GTTH, OWJ, and MS), in which each code was characterised by a description, inclusion and exclusion criteria, and typical and atypical exemplars to guide the coding process. To ensure coding consistency among the study team members, one transcript was first coded to establish inter-rater reliability. The study team continued to discuss, refine the codebook, and repeat the coding with another transcript until a satisfactory inter-rater reliability score was achieved (Cohen’s Kappa score > 0.75). Transcripts were then distributed among the study team members for coding. Data analysis was completed with Nvivo Version 11.0. (QSR International Pty Ltd. Hawthorn East, Australia).

After coding all transcripts, the codes were sorted such that similar codes were grouped together to form potential themes. Codes that did not seem to fit into any theme at first were revisited as the themes were gradually refined. Relationships between these themes were also examined, and different levels (main theme and sub-themes) were identified. Any remaining codes were compared against the revised themes to determine fit. The initial themes were drafted by SS, JCMG, GTTH, OWJ, and MS and presented to CSA for further refinement before finalisation.

Strategies to ensure the quality of the findings recommended by Anney [46] were exercised in this research. Data were triangulated from two different informant sources: patients and caregivers. The transcripts were read and re-read by five researchers independently. The interpretations were compared, and regular meetings were held to discuss differences until a consensus was reached. These informant and researcher triangulations aimed to increase the credibility of the findings. To ensure transferability, the participants were sampled in such a way that there was good distribution by age, gender, education level, and ethnicity, and for the caregivers, relationship with the care-recipient. Finally, for confirmability, intentional record keeping of summaries and reflections after each FGD as well as decisions made during the coding and analysis were documented to maintain an audit trail.

## 3. Results

Four overarching themes depicting the strategies to combat stigma were identified. They included (1) raising mental health awareness, (2) social contact, (3) advocacy by influential figures or groups, and (4) legislation of anti-discriminatory laws. It was not uncommon for participants to refer to two or more approaches in a single quote. While we have selected quotes to illustrate the main theme, they may cross-cover other themes to some degree. To ensure that standard usage of English was maintained, minimally corrected verbatim of quotes are presented.

### 3.1. Raising Mental Health Awareness

There were two subthemes pertaining to the strategy of raising mental health awareness, which can be described as the “who and how” and “what” of this approach. 

#### 3.1.1. Target Groups/Setting and Methods

Anti-stigma awareness initiatives for the general population were frequently suggested by participants, and they recommended outreach through both traditional and social media as well as popular mass events such as marathons and festivals in order to reach a wide range of members of the public from the young to the old. They also emphasised that these efforts should be carried out repeatedly, reasoning that increased exposure to the topic will lead to greater familiarity and with time, greater acceptance of this taboo subject.

*You all have to do a lot of campaigns, running it tends to stick in their minds (Male, 37 years, Psychosis-related disorder, PWLE FGD 5)*

*Educating the public because it is very important. More on media because there are many people on the internet or computer, TV and all sorts ah, newspaper of course, articles, so that more people will come to know so that lesser, I mean to accept slowly. The stigma will grow weaker and weaker, not that strong. (Female, 65 years, Caregiver FGD 3)*

With regards to media portrayal, the participants discouraged the use of drama in and of itself as a means of education. They asserted that such media forms tend to sensationalise mental illness through negative characterizations that further reinforce existing stereotypes. Instead, they prefer coverage through documentaries and talk shows.

*I think drama is not a good way to go to raise awareness of mental illness because in drama, it’s drama what. You have to be dramatic, and you have to be dramatic you have to sensationalise. But maybe talk shows are better. Talk show where they invite celebrities together with professionals and then they talk it out. (Male, 28 years, Psychosis-related disorder, PWLE FGD 5)*

Raising mental health awareness in two specific target groups was frequently brought up. The first group that the participants wanted to increase mental health awareness in was among school students. The participants rationalised that it would be easier to influence young people whose attitudes are more malleable. They also voiced concerns that youth are a vulnerable group due the heightened challenges they face such as intense academic pressures and societal expectations. Thus, they would benefit from literacy-based interventions to facilitate early recognition and help-seeking behaviours. Some of the participants suggested an incremental approach whereby younger primary school-age students could be primed with information on mental well-being before being introduced to more serious topics on mental health conditions.

*Education from, it’s ok for those who are already old enough like us, we can’t like, some people we can’t really unlearn what we learn so we have to teach the new generation and input in what are the more important things, like for example, the major concerns as of now and like how important is mental health so that they won’t grow up to be like their parents or like whoever. So, I think it starts from the younger ones la they will receive fresh information and they won’t have any judgement against us (Female, 22 years, Mood disorder, PWLE FGD 2)*

*I think it should start across Singapore, like for example, starting from schools, starting from workplaces, meeting the employers you know, the teachers, the, the facilitators, starting from there you know. So, when you have that well-established, designed activities you know, I mean the initiative, we have that initiative to go to, to the schools you know, at a younger age when they already start to understand, so when they come to work, as an maybe an employer, so they can understand further what happens to the subordinates, what happens to the employees who have mental illness. (Female, 48 years, Caregiver FGD 5)*

The second target group was employers. The participants commented that in contrast to the recent advances in efforts to raise mental health awareness, workplaces were especially lagging in this regard, and overt stigmatisation continues to occur in workplaces. Legislative measures were also recommended to combat stigma and discrimination in this setting (this will be further elaborated in the fourth theme).

*Essentially run multiple small campaigns across companies so we start from the top tier and then you go down to the middle tier and then into the SME like a small, very small enterprises and things like that to do awareness campaign with all their HR [Human Resource], their ops [operations] department and things like that so that they learn. So at least if the managers learn, hopefully they’ll pass that on, not always. It will not always happen, but it might you know, so at least there’s a trickle-down effect and it last longer (Male, 28 years, Mood disorder, PWLE FGD 6)*

*I think the Singapore government is very effective, and they are respectable, so Ministry of Health or IMH organize talk to the employers, to the unions, trade unions. (Male, 64 years, Caregiver FGD 5)*

#### 3.1.2. Types of Content

The participants recommended that messages pertaining to the prevalence of mental illness debunking common myths about mental illness and likening it instead to other chronic illnesses could reduce stigma and demystify mental illness. The participants also recommended disseminating information on where and how mental illness can be managed as well as the efficacy of treatments to emphasise the treatability of mental illnesses.

*Get the MP [member of parliament] come and talk, share with them, share with the population, mental illness is common. Especially depression, 1 in 5, in the population will get depression, or even now, now 1 in 4. (Male, 64 years, Caregiver FGD 5)*

*To me education is the best way to, to erase all these myths (Male, 49 years, Psychosis-related disorder, PWLE FGD 4)*

*Let them know, no big deal. It is just like any other chronic illness. I say in your whole lifetime anyone can suffer some from of mental illness. (Female, 65 years, Caregiver FGD 3)*

The PWLE cautioned that anti-stigma messages should not result in “over-normalisation” or careless over-identification of mental illness and trivialisation among lay members of the public, as it makes light of the disruptions to the lives of those who have been diagnosed with them and the suffering they bring.

*You don’t want to reduce stigma so much to the point when somebody likes oh I like to put my water bottle on the right side, oh I’m OCD. You know. Or like oh I had a negative thought that came in, oh I have schizophrenia. You don’t want people to like over-normalise it, cause that does happen in these days. Like when just somebody likes to be neat, they consider themselves OCD. Yeah, it’s so frustrating. The definition of mental illness is it’s supposed to disrupt your natural life. If it doesn’t disrupt your life in any way, it’s not supposed to be considered a mental illness. So, when people are like oh I’m very OCD, or like oh I have depression. Yeah, everybody has depression. (Female, 23 years, Psychosis related disorder, PWLE FGD 5)*

They also did not wish for others to take pity on them or treat them differently on account of their mental illness but to instead be supportive in their recovery.

*You shouldn’t get them to sympathize with you, more like to understand them. Rather than like say that okay you must be careful of this people, you must just give them information you know (Male, 24 years, Mood disorder, PWLE FGD 6)*

*We would educate them on how to less stigma, how to know about our condition, then how to actually support us in the recovery process (Female, 34 years, Psychosis-related disorder, PWLE- FGD 4)*

### 3.2. Social Contact

Three kinds of social contact were raised by the participants: celebrity disclosures, testimonies of success stories by people with mental health conditions and opportunities to interact with them.

#### 3.2.1. Celebrity Disclosures

The participants exemplified that the disclosure of mental health struggles by local celebrities as being particularly impactful. They reasoned that Singapore has a celebrity culture in which celebrities have a large following and influence. Sharing their mental health difficulties would drive the message that mental illness does not discriminate, debunk certain stereotypes associated with mental illness, validate the experiences of those coming to terms with their illness, and encourage open and honest discussion about mental illness.

*I think it’s good if you can get someone, well known in Singapore, to share about their family members having mental illness or they themselves having mental illness. (Female, 51 years, Caregiver FGD 5)*

*In Singapore, there is a podcast. That is actually supposed to be a comedy podcast, but every now and then it gets a bit real. And they talk about themselves. So, I think, I don’t know if you guys know Nathan Hartono? He runs a podcast with his friend named Jon Kensey who’s also based in Singapore. He’s Filipino but he’s based in Singapore. He does comedy shows, he does, he’s a comedian. He does all these funny little things but every now and then when we write in, and we are allowed to write in to them it’ll just be like an idea of what would you suggest if we are going through this thing. So, he actually openly talks about his depression. So, he’ll share experiences, he’ll share what he would do, he would make things very clear that it’s not supposedly the only way or the best way to do things but that’s how he would have done it. Yeah, and the thing these little things help knowing that even people who are supposedly seen on a higher pedestal is also facing the same things as you are. Yeah, really changes a lot (Male, 23 years, Mood disorder, PWLE FGD 1)*

#### 3.2.2. Sharing Inspirational Recovery Stories

Apart from celebrity figures, the PWLE suggested that inspiring recovery stories of people with lived experiences can be included in anti-stigma efforts. They reasoned that these stories illustrate that mental illness does not need to be a barrier to attaining a meaningful life, that people with mental health conditions could be productive members of society, an embodiment of strength and courage despite adversity, and give hope to those worried about their or their loved one’s future.

*I remember that time I was watching the news about lady, she suffered from mental illness but she… she see doctors, follows up regularly, she is recovered in that sense. She secured a job as a lecturer in one of the local polytechnic. She wrote a book. She was very frank with her employer, that was good, and they accept her as she is but she still go back to the hospital like every several months for follow-up and stuff like that. So I find that very enlightening. That there is such people who actually like make good head way in their life despite having mental illness. So I think if you can find more of such people and interview them highlight to the public that is actually… we can also be successful in their own way, it will be good. (Female, 44 years, Psychosis-related disorder, PWLE FGD 3)*

#### 3.2.3. Opportunities for Social Contact

The participants opined that it would be beneficial for those without mental illnesses to have opportunities for direct social contact with PWLE. They identified the benefits of this strategy as allowing people to relate to PWLE on a more personal level and debunk extreme examples of mental illness. They added that such a first-hand experience would be more compelling than didactic approaches.

(*Another participant: Education is important) And exposure, I think. Because education is like you are telling people you know? But I think exposure for them to experience it, interaction with people with mental illness. It really speaks more than words. How they… because people always have this misconception ‘oh ok, mental illness, violence, negative, crazy, talking to yourself…’ But in fact, in fact most mental patients are not like that, they don’t behave that way. (Female, 39 years, Caregiver FGD 1)*

*I would emphasize priorities on interaction between the regular population and those with illness. To let them realize there’s no big difference. (Male, 24 years, Mood disorder, PWLE FGD 6)*

### 3.3. Advocacy by Influential Figures or Groups

The participants felt that the question of “who” leads the de-stigmatisation efforts matters. Some participants suggested that mental health experts should partner with organisations that have larger influence, while others asserted that the efforts should be helmed by organisations other than psychiatric experts.

*Because like even in my head, the assumption is it’ll probably come from IMH. Which to me is not the most effective method of campaigning because then everybody would be like, yeah they would do this, of course they would do this. So, I think, no. (Male, 23 years, Mood disorder, PWLE FGD 1)*

They identified individuals and groups with political affiliations as being particularly suited due to their authoritative influence, networks, and access to funds.

*To be effective it has to be nationwide, government-led initiative. It cannot be a little bit here and there, by IMH or by Caregiver Alliance, it doesn’t really work. It has to be nationwide and it has to have the support of the government. First, they have the resources and as what (another FGD participant) said uh, they can be very effective if they are very serious in wanting this campaign to be successful, they can do it. (Female, 51 years, Caregiver FGD 5)*

### 3.4. Legislation of Anti-Discriminatory Laws

#### 3.4.1. Removal of Declaration of Mental Illness in Job Application and Scholarship Forms

In virtually all the FGDs, the participants called for the removal of the declaration of mental illness from job application and scholarship application forms. They regarded this requirement as being both irrelevant and discriminatory and believed that their unsuccessful applications were directly due to their disclosure.

*Now we talk about the functional ability of a mental illness person, we talk about work. When you work, you need to fill in a lot of forms. There are a lot of things that…why must there be a declaration by the organisation? Why? Why? That is not good, that is not fair. In school, in army or in certain big organisation, there is always a declare, “Are you mentally ill? Do you have a mental problem?” Why is that in the form? It should be out. (Male, 62 years, Caregiver FGD 1)*

*I hope like whenever you’re applying for a job then you do not need to tick off the, “Do you have a mental illness?” that... that column. But maybe being open about it and talk about it would make the person understand in your shoes (Female, 25 years, Mood disorder, PWLE FGD 6)*

#### 3.4.2. Policies That Encourage Employers to Hire and Support PWLE in Workplaces

Although the participants called for the removal of mental illness declarations on application forms, they preferred to be truthful about their condition, as they feared being discovered if they had lied to increase their chances of employment. They also found that concealing their condition and their medical appointments was burdensome. The PWLE suggested that apart from raising awareness among employers, governmental support could be given to encourage employers to hire individuals with known mental health conditions.

*The government could perhaps encourage their employers to take on mental patients who are willing to disclose their conditions of their job contracts. I stay in (name of a sheltered home) and the practice of their employment specialist is to have a very honest declaration. Just help… just say we have, this person who is going to help work has a mental condition. So they say that is why I follow them because they do it on a clean cut basis but I feel very upset because I follow suit, I declare my condition, and I loss so many chances of being employed. So government can do, I think government has yet to, have a lot to do. (Female, 55 years, Psychosis-related disorder, PWLE FGD 3)*

*In line with my unemployment, no money, and at the same time, I feel like I can contribute la to the greater society, ah you know what I’m saying? So that maybe in the future, people will say okay never mind you can work but then like you know, every year got psychiatric evaluation you know that’s even better, you know. A company that’s accepting of your condition. (Male, 24 years, Mood disorder, PWLE FGD 6)*

Lastly, several participants shared being caught in awkward situations upon disclosure and the found reactions by their superiors inappropriate. Thus, they suggested that guidelines can be put in place to inform employers on how to respond sensitively to those who choose to disclose.

*Maybe you can suggest to MOM [Ministry of Manpower] to remove the questions about health conditions. Have some regulations or advice to employers about how they can react to people who declare or maybe even take it out from the application form. For example, in the US [United States], you’re not actually, it’s kind of discriminatory, you’re not allowed to. It’s against the law to discriminate people based on their orientation or whatever, their conditions. So maybe in Singapore, we can move ahead in that regard. (Male, 28 years, -Psychosis-related disorder, PWLE FGD 5)*

## 4. Discussion

The suggestions by the PWLE and caregivers in tackling stigma can be classified broadly as raising awareness through education, social contact, advocacy, and legislative reform. These strategies are in line with the approaches that have been used internationally to disrupt the process of stigma. Corrigan et al. [47] have suggested three approaches: education, protest, and contact, while Arboleda-Florez and Stuart [22] extended Corrigan’s typography with three additional strategies: legislative reform, advocacy, and stigma self-management. Our study has further identified nuanced details on how this can be carried out in the Singapore landscape.

Raising awareness through public education was the most suggested strategy. It is appealing, as it targets lack of awareness and misinformation with the provision of information. Young people in particular were identified as a target group for educational intervention due to the burgeoning pressure they face, the potential they have in changing the future, and the opportunities that school settings have to deliver interventions using a literacy approach, points that have also been described previously [48,49] in the literature. School-friendly literacy approaches have been shown to be effective in improving knowledge about mental health conditions [50].

The participants suggested various ways of raising awareness for the general population and drew particular attention to the potential problems of media strategies due to their tendency towards presenting stories in a sensational manner. Gottipatti et al. [51] explored local media portrayals of people with mental disorders in Singapore’s largest media organisations, Singapore Press Holdings and MediaCorp, and reported that mental health-related articles in Singapore were primarily negative in sentiment, with crime-related news accounting for 40% of the corpus. Moreover, mental health experts had unwittingly used stigmatising terms in interviews. The authors suggested that media professionals and editors can also be enlisted to play a more proactive gatekeeping role and to counteract the largely negative portrayal of mental illness by providing articles on well-being and recovery. They also proposed a rule-based solution model based on text mining and natural language processing (NLP) techniques that can automatically identify aspects of stigma in media articles for editing before publication [51]. Similarly, Stout, Villeges, and Jennings [52] suggested for informational and educational activities to be arranged for journalists that teach techniques to report on mental health stories in a balanced and responsible way. Other strategies that have been used include SANE Australia’s (a charitable advocacy group) StigmaWatch program, which monitors and responds to the inaccurate or inappropriate media portrayal of mental illness. If problematic reporting is not remedied, then the group may consider taking public action [35]. 

The participants also cautioned that messaging should not result in benevolent stigma—an unintended consequence of well-intentioned approaches, where people with mental illness are treated as being unable to competently handle life’s demands and need a benevolent authority to make decisions for them [30]. This kind of messaging is stigmatizing, as is perpetuates power imbalances by appealing to the public to do good for a seemingly weak, pitiful subordinate group [53]. Such a response disempowers people with mental health conditions and stunts opportunities for personal growth. The participants in our study, as Corrigan had pointed out, wished for empathy cultivation leading to being treated with parity rather than pity. They also cautioned that efforts at normalising mental illness should not result in the trivialisation of the disorder. Research has suggested that those who accept their mental illness as part of their identity, overcome the challenges of stigma, and who remain resilient, may feel a sense of pride in being able to do so [30]. Thus, symptoms that are “undeserving” of the diagnosis may be viewed as discrediting their strength and authenticity. 

The second theme that the participants identified was an opportunity for members of the general public to have social contact with a PWLE. They believed that many members of the public had never had personal interactions with a person with mental illness and formed attitudes towards people with mental health conditions based on stereotypes. In line with Allport’s [54] intergroup contact strategy, they proposed that personal contact with individuals with mental health conditions would reduce prejudice towards them. Purely naturalistic contact in everyday settings, however, does not reduce stigma [55], as high rates of stigma persist amongst mental health service staff who are in contact with individuals with mental health conditions on a daily basis [56]. Effective contact-based strategies are planned interactions with certain conditions in place. Some of these conditions relate to the credibility of the speaker, the local relevance to the audience, the disclosure of personal struggles due to the illness and “on the way up stories” describing successes in areas of living independently, employment and in having quality relationships [21,25,31,57] A local study examining the impact of a combined education and face-to-face contact intervention with university students showed that the intervention significantly improved stigma, reduced the desire for social distancing, and improved attitudes towards help-seeking [32,33,58]. However, the effects of this intervention was short-term, highlighting our participants’ assertion as well as supporting literature that stigma-reduction efforts should be continuous [57].

While such planned interactions can be logistically challenging and encumber continuity of exposure, the evidence suggests that non-face-to-face contact can also deliver notable results [59]. Schiappa et al.’s [60] “parasocial contact theory”, which was built upon Allport’s hypothesis proposes that sustained mass-mediated contact with a media figure engenders a parasocial relationship where the viewer feels a real, emotional connection with the figure that is akin to that with a close friend. Thus, the revelation of a stigmatised identity from the celebrity with whom a parasocial bond already exists has a greater potential to reduce stigma than an encounter with someone new who reveals the same stigmatised identity [61]. Indeed, an experimental study among undergraduate students showed that exposure to a video of popular pop singer, Demi Lovato, disclosing her experience with bipolar disorder (BD) significantly reduced negative stereotypes towards BD, and the higher the level of parasocial relationship, the lower their negative stereotypes of people with BD were [59]. There is also potential for repeated exposure to mass-mediated content to effect long-term change, as demonstrated in a longitudinal study in which college students who were presented with filmed social contact every 2 months over a 12-month period showed significantly positive long-term outcomes (24 months follow-up) in terms of their behavioural intentions for social contact with PWLE compared to the control groups [62]. Participants highlighted that this approach could serve well in a society such as Singapore, which has a celebrity culture. 

The third and fourth themes relate to advocacy by influential figures/groups and the legislation of anti-discriminatory laws. Legislative reform is designed to prohibit discrimination on any grounds, improve the protection of people with mental illness, and offer reasonable accommodations in areas such as employment, education, and housing. Advocacy is designed to ensure that people with mental illness enjoy the rights and freedom offered by legislation and provide avenues of redress for inequitable policies and procedures [22]. Discussions among participants on these themes highlighted the importance of governmental organisations, grassroots leaders, and other public figures who carry the clout to push for changes in policy and practices to create an environment that is more inclusive and supportive of people with mental health conditions. They argued that mental health experts are not the best people for this task, alluding to the fact that it could be perceived as an act of self-interest to promote the importance of their profession—a point also identified by Clement et al. [63]. Corrigan [30] contrasted the service agenda in targeting stigma with the rights agenda, where the former is aimed at removing barriers to professional care, while the latter replaces disenfranchisement with affirming attitudes and behaviours. These agendas, which have different purposes and processes, may compete, and choices need to be made in allocating resources to work towards the chosen endpoint [30].

The participants in the study commented that mental health awareness has increased in Singapore in the past couple of years, but these advancements have not caught on as quickly, particularly in workplaces. A significant proportion of PWLE remain unemployed despite their desire and ability to work [64]. At the time when the data were collected, mental illness declarations were still a part of job application processes. In the FGDs, the participants zealously urged for this practice to be put to an end, as it impeded their employability and career ambitions. Further to the removal of this declaration, they suggested that support schemes should be offered to employers for hiring people with mental illnesses. A systematic review of anti-stigma interventions in workplaces showed that targeted interventions could lead to improved employee knowledge and supportive behaviour towards people with mental health problems [65]. However, due to the methodological shortcomings of the included studies, the heterogeneity of the intervention content and other issues, no efficacious intervention element could be identified. The current evidence suggests that specific workplace messages are more influential than more general workplace messages, and these messages should also be tailored for different stakeholders within the workplace (e.g., supervisors vs. employees) [66]. Corrigan recommended that identifying specific stigma change goals can be undertaken through a needs assessment conducted together with the target group [67]. One component of stigma change identified in this study was the inclusion of practice guidelines on responding to disclosure appropriately, fairly, and sensitively in workplaces.

Several mental illness de-stigmatisation endeavours, some of which have incorporated the suggestions identified in this research, have occurred in Singapore. First, Singapore launched its first long-running anti-stigma campaign called Beyond the Label in September 2018, with PWLE fronting the campaign and a strong social media presence. In January 2019, the first insurance policy that covers common mental illnesses, the AIA Beyond Critical Care, was launched by AIA Singapore. In December of the same year, the Tripartite Alliance for Fair and Progressive Employment Practices declared that asking job applicants about their mental health condition without good reason was discriminatory and that employers who do not abide by the updated guidelines may be liable to enforcement actions. As one of the PWLE in our FGD summarised,

“It’s moving, it’s slow…but it is gaining momentum…This is the best time for the mentally ill. People are more able to speak up for themselves even as patients and that is a wonderful thing”.

There are a few limitations to this study. First, the participants were recruited on a voluntary basis. Thus, the findings may not reflect the broader views of PWLE and caregivers. Two FGDs had only four and five participants, which may have made the dynamics of that group different from the larger-sized ones although the analyses did not suggest any specific themes coming up or being omitted in those FGDs. Second, as the researchers were from the IMH, participants have may withheld their critical views of the mental health system and suggestions to improve it, as it was possible that they did not want to offend the researchers affiliated with the institution. Third, the data were analysed from the lens of the researchers—the PWLE and caregivers were not part of the team analysing the data. 

Notwithstanding these limitations, this study is one of a few studies in Singapore to have obtained inputs on stigma reduction from the perspectives of those who have been stigmatised. While several positive steps have been taken towards de-stigmatisation, evaluation efforts for these initiatives are currently lacking. It is recommended that scientifically rigorous evaluations of these efforts be undertaken so that outcomes can be tested, and the initiatives can be continually improved. Further, PWLE should be included in future anti-stigma research as well as in the evaluation of these campaigns and programmes.

## 5. Conclusions

The PWLE and caregivers highlighted that careful deliberation is needed when crafting messages to raise awareness about mental illnesses to avoid the unintended effects of trivialisation of these illnesses and the generation of sympathy. The research also identified how individuals such as community leaders and media figures are in advantageous positions to reduce stigma and advocate for better support for people with mental health conditions. The continued evaluation of new anti-stigma strategies is important to informing the impact of these efforts as well as to fine-tune future initiatives.

## Figures and Tables

**Table 1:** Sociodemographic characteristics.

	PWLE	Caregiver
	Mean (Range)	Mean (Range)
Age (years)	33.4 (21–58)	53.5 (22–73)
	N = 42	N = 31
Sex		
Male	18	10
Female	24	21
Highest Completed Education ^#^		
Secondary education and below	7	9
Vocational certification/Diploma/Pre-U	26	14
University degree and above	8	8
Ethnicity		
Chinese	27	21
Malay	10	4
Indian	4	5
Others	1	1
Illness type		
Mood	18	
Psychosis	24	
Relationship with care recipient		
Spouse		2
Parent		15
Child		8
Sibling		4
Others		2

^#^ 1 missing PWLE response for Highest Completed Education.

## Data Availability

The datasets generated and/or analysed during the current study are not publicly available due institutional policy but are available from the corresponding author upon reasonable request.
